# State Based Model of Long-Term Potentiation and Synaptic Tagging and Capture

**DOI:** 10.1371/journal.pcbi.1000259

**Published:** 2009-01-16

**Authors:** Adam B. Barrett, Guy O. Billings, Richard G. M. Morris, Mark C. W. van Rossum

**Affiliations:** 1 Institute for Adaptive and Neural Computation, University of Edinburgh, Edinburgh, United Kingdom; 2 Centre for Cognitive and Neural Systems, University of Edinburgh, Edinburgh, United Kingdom; UFR Biomédicale de l'Université René Descartes, France

## Abstract

Recent data indicate that plasticity protocols have not only synapse-specific but also more widespread effects. In particular, in synaptic tagging and capture (STC), tagged synapses can capture plasticity-related proteins, synthesized in response to strong stimulation of other synapses. This leads to long-lasting modification of only weakly stimulated synapses. Here we present a biophysical model of synaptic plasticity in the hippocampus that incorporates several key results from experiments on STC. The model specifies a set of physical states in which a synapse can exist, together with transition rates that are affected by high- and low-frequency stimulation protocols. In contrast to most standard plasticity models, the model exhibits both early- and late-phase LTP/D, de-potentiation, and STC. As such, it provides a useful starting point for further theoretical work on the role of STC in learning and memory.

## Author Summary

It is thought that the main biological mechanism of memory corresponds to long-lasting changes in the strengths, or weights, of synapses between neurons. The phenomenon of long-term synaptic weight change has been particularly well documented in the hippocampus, a crucial brain region for the induction of episodic memory. One important result that has emerged is that the duration of synaptic weight change depends on the stimulus used to induce it. In particular, a certain weak stimulus induces a change that lasts for around three hours, whilst stronger stimuli induce changes that last longer, in some cases as long as several months. Interestingly, if separate weak and strong stimuli are given in reasonably quick succession to different synapses of the same neuron, both synapses exhibit long-lasting change. Here we construct a model of synapses in the hippocampus that reproduces various data associated with this phenomenon. The model specifies a set of abstract physical states in which a synapse can exist as well as probabilities for making transitions between these states. This paper provides a basis for further studies into the function of the described phenomena.

## Introduction

It is widely believed that synaptic potentiation, as demonstrated by the physiological phenomenon of long-term potentiation (LTP), plays an important rôle in memory formation in the brain [1],[2]. This has triggered a vast number of experiments in which this phenomenon has been recorded, both *in vivo* and *in vitro*. Typically, LTP can be elicited in a population of CA1 neurons by placing an electrode into an input pathway in the stratum radiatum, and applying a burst of high-frequency stimulation.

One major result that has emerged is that there are at least two distinct “phases” of LTP, see [3] for a review. Firstly, there is an “early”, transient phase (e-LTP) that can be induced by a single, brief (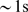), burst of high-frequency stimulation (weak HFS). The lifetime of this phase is around three hours in slice experiments, and its expression does not require protein synthesis [4]–[6]. Secondly, there is late-phase LTP (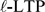), which is stable for at least the eight hour time-span of a typical slice experiment, but which can last up to months *in vivo* [7]–[9]. 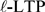 can be induced by repeated (typically three) bursts of HFS, separated by 10 minute intervals (strong HFS). Thus, notably, more stimulation does not increase the amount of synaptic weight change at individual synapses (as often assumed in models), but rather increases the duration of weight enhancement. It has been shown that protein synthesis is triggered at the time of induction and is necessary for 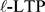 [4],[5], although a more complicated rôle for protein synthesis in LTP has been implied [10],[11].

Interestingly, e-LTP at one synapse can be converted to 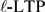 if repeated bursts of HFS are given to other inputs of the same neuron during a short period before or after the induction of e-LTP at the first synapse [12]–[14]. This discovery led to the hypothesis that HFS initiates the creation of a “synaptic tag” at the stimulated synapse, which is thought to be able to capture plasticity-related proteins (PRPs). The PRPs are believed to be synthesized in the cell body, although recent data suggest they may be manufactured more locally in dendrites [15]. The general framework for these hetero-synaptic effects is called “synaptic tagging and capture” (STC). Which proteins are involved in each stage of STC has not been fully elucidated yet. Current data suggest that, at least in apical dendrites, calcium/calmodulin-dependent kinase II (CaMKII) is specifically involved in signaling the tag in LTP induction [15] and protein kinase 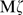 (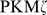) is involved in the late maintenance of potentiated synapses [6],[16].

The counterpart of LTP, long-term depression (LTD), can be induced by stimulating CA1 hippocampal neurons with low-frequency stimulation (LFS) [17],[18]. LTD states appear to have analogous properties to the LTP states discussed above. The early phase, which we call e-LTD, lasts around three hours, is not dependent on protein synthesis, and can be induced by weak LFS, consisting of, for example, 900 stimuli at 1 Hz. For induction of the late phase, 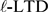, a stronger form of LFS is required, for example 900 bursts of three stimuli at 20 Hz, with an inter-burst interval of one second [19]. Like 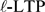, 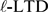 is stable for the duration of most experiments and is protein synthesis dependent [20]. Moreover, e-LTD of one synapse can be converted to 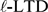 if strong LFS is given to a second synapse of the same neuron within an interval of around one hour [19]. The setting of LTD tags appears to be mediated by mitogen-activated protein kinases [15], but no 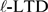 specific PRP is yet known.

It turns out that LTP and LTD are not independent processes and that an interaction known as “cross-capture” can occur between synapses tagged for LTP and synapses tagged for LTD [19]. Thus 1) e-LTD of one synapse can be converted to 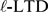 by giving 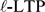 inducing strong HFS to a second synapse shortly before or after the induction of e-LTD at the first synapse; 2) e-LTP can be converted to 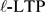 in an analogous manner. Cross-capture suggests that strong HFS and strong LFS both trigger synthesis of both 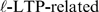 proteins and 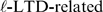 proteins.

A separate strand of research has put forward the idea that plasticity protocols cause synapses to make discrete jumps between weak and strong states [21],[22]. Discrete synapses have a number of interesting theoretical properties, for example: 1) old memories become at risk of being erased as new ones are stored, (e.g. [23]); 2) synaptic saturation, important in preventing run-away activity, is automatically included, while storage capacity can be high [24].

There have been several biochemical models that posit binary synapses [25]–[31]. Induction and maintenance of activity-dependent plasticity has been successfully incorporated into a recent study [31], and the longevity of evoked synaptic changes has been investigated [28],[29]. There is however great divergence between most network-level plasticity models and the experimental observations outlined above. Network models typically ignore interaction between synapses, use graded weights, and assume that the stimulus only determines the amount of weight change and not its longevity.

Given the limited knowledge of the processes involved, a detailed model seems at present out of reach. Instead, the model we present in this paper aims to integrate the key results from experiments on induction, maintenance and STC together into a concise model, whilst remaining simple enough to be useful for neural network modeling. The model posits a set of possible physical states in which a synapse can exist, including, in particular, states with a tag present. The states are characterized by their synaptic strength, and also by their resistance to potentiation and depression. These characteristics are assumed to be determined by the number of AMPA receptors present in the membrane [32], and by the configuration of proteins within the post-synaptic density (PSD) [33]. In our model, a synapse existing in one state will evolve by making stochastic transitions between the different states, the probability per unit time of any given transition being specified explicitly by the model. High- or low-frequency stimulation is assumed to change these transition probabilities.

The model does not, at this stage, include the complete biochemical machinery involved in the induction, expression and maintenance of synaptic plasticity. Instead, for reasons of computational efficiency, we develop a high-level model that abstracts these processes and concentrates on the quantities important for network behavior, namely the induction protocols and the resulting weight changes. The model reproduces sufficient agreement with real data to render it useful in exploring further the functional consequences of STC in network modeling.

## Methods

### Simulating Electrophysiology Experiments

We have used our model to simulate several electrophysiology experiments with multiple populations of synapses. More specifically, we consider stimulation of multiple independent synaptic inputs to the same neuronal population in CA1, such that a protein synthesis-triggering stimulus (i.e., strong HFS or strong LFS) to one input affects all populations of synapses, and leads to STC interactions between populations. The stimulation protocol for the experiment sets the transition rates for synaptic state transitions within each population.

In all cases we assume that at time 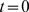 there have been no recent stimulation protocols, and that the system is in equilibrium. Thus, initially, all transition rates are at their resting values, and all synapses occupy one of the basal states. Moreover, within each population, 80% of the synapses occupy the weak, as opposed to the strong, basal state (see Results). Note though, that in a real experiment, not all synapses will be in basal states, because they might have experienced strong stimuli already, earlier in life. As a result, some synapses may already be in the 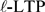 or 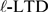 state before the experiment is started. These will however remain in these states throughout the experiment, and not interfere with other synapses, so they can be ignored. However, the presence of such synapses would reduce the observed amount of LTP/D, both in model and experiment.

**Figure 1:**
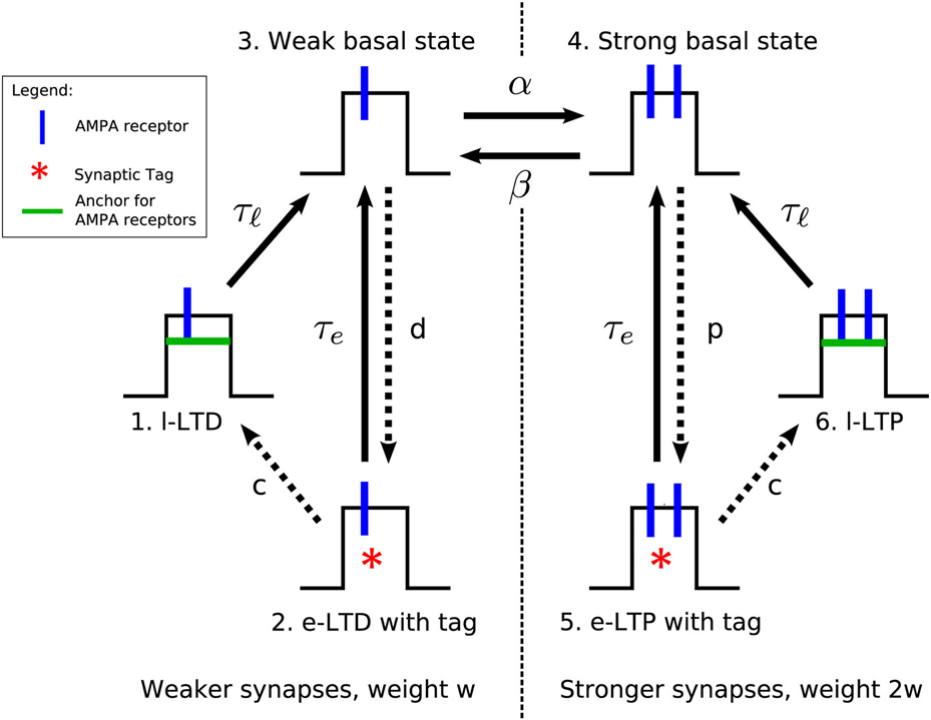
Diagram of synaptic states in the model. The three states on the left have weak weights, whereas the three states on the right are strong. Arrows indicate the transitions, whilst the symbols next to the arrows denote transition rates. The dotted arrows indicate transitions that are only active after a plasticity-inducing stimulation. In addition, all transition rates except those labeled 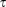 change when a stimulation protocol is given (see text for details). In the absence of recent stimuli, values of the transition rates are 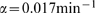, 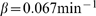, 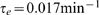, 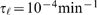, 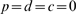. In that case the synapse fluctuates between states 3 and 4. Note that the drawing of weak states with a single AMPA receptor and strong states with two AMPA receptors is intended to be merely a figurative rather than precise illustration; similarly with the “anchors”.

The actual number of synapses measured in experiments using extra-cellular recordings is not known and probably varies considerably between experiments. The results we obtain come from taking 1000 synapses in each population. Starting from the initial equilibrium condition, we update state occupancy numbers at each time-step by random sampling in accordance with the transition rates. Then, for each population 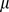, we can find the relative field excitatory post-synaptic potential 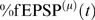 by expressing the summed synaptic weight at time 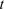 as a percentage of the initial summed synaptic weight:
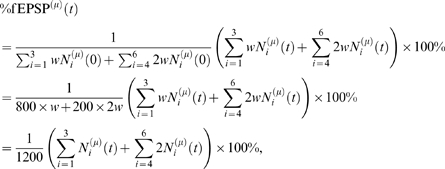
(1)where 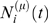 denotes, for population 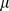, the occupancy number of state 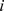 at time 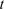, with the states numbered as in Figure 1. Furthermore, we used that the weight of states 4,5,6 was 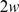, twice that of states 1,2,3.

### Mean Weight Change and Its Fluctuations

In addition to stochastically simulating experiments, it is possible to calculate mean results as well as the inter-trial standard deviation for each experiment we simulate. Let us consider a single population of synapses within an experiment. Let 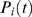 denote the probability that a particular synapse is in state 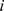 at time 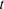. Then the time evolution equation for the 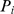 is given by
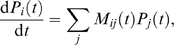
(2)where the matrices 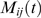 are defined by
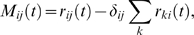
(3)and 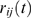 denotes the transition rate from state 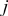 to state 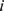 at time 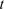 (with the convention that the 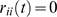). Using equations (2) and (3), and the fact that at all times the occupancy numbers 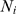 follow a multinomial distribution with parameters 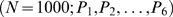, it is straightforward to obtain the following equations for the moments of the 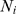:
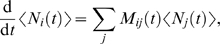
(4)

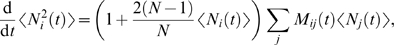
(5)

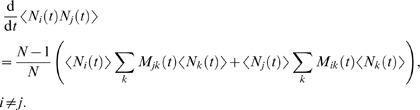
(6)From these equations, together with equation (1), we obtain
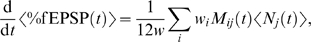
(7)

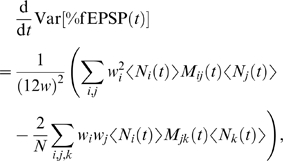
(8)where 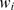 is the weight associated with state *i*, 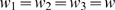, 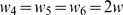. Numerical integration of equations (4), (7) and (8), from appropriate initial conditions, enables us to plot the mean and the standard deviation of 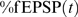. Using the equilibrium multinomial distribution 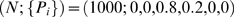, the appropriate initial conditions are 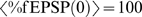 and 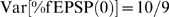.

## Results

### Description of the Model

Our model is designed to reproduce as much pre-existing electrophysiological data on long-term plasticity and STC as possible, whilst at the same time remaining as simple as possible for its purpose. In drawing up a list of states, a trade-off must be made between having few states and complicated transition rate dynamics or having lots of states and simple transition rate dynamics. Our convention is to say that states are distinct if they differ either in their synaptic strength or in the expected time it will take them to potentiate or depress in the absence of any plasticity protocols. This leads us to a six state model, containing three weak and three strong states: weak basal, strong basal, e-LTD, e-LTP, 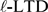 and 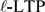. The reactions that are triggered by plasticity protocols are incorporated via time-variable transition rates between these six states.

Figure 1 shows schematic drawings of the synaptic states of the model, together with the allowed transitions between states. The rate parameter associated with the transition from one state 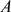 to another state 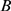 gives the probability per unit time of a synapse in state 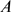 making the transition. Equivalently, the inverse of the rate parameter is the average time it takes the synapse to make the transition (assuming no other transition is available). In our simulations we model populations of synapses, with each individual synapse behaving independently with respect to making transitions between states. In mathematical terms, our model is a stochastic Markov process. Effects of stimulation protocols are modeled by transient changes to the transition rates. To model STC, certain stimulation protocols given to just one population of synapses can affect the transition rates of multiple populations. These hetero-synaptic effects reflect the capturing component of STC.

### Description of the Six Synaptic States

In the absence of stimuli, synapses fluctuate between a weak and a strong basal state. The weak basal state is assigned an arbitrary synaptic weight 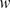, whilst the strong basal state is taken to have synaptic weight 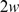. These could correspond to the two states probed in the experiments of Ref. [22], in which it was found that the pairing of a brief steady current injection with an appropriate depolarization led to switch-like approximate doubling or halving of synaptic efficacy. The difference in efficacy between the two states is assumed to come about from AMPA receptor insertion/deletion. The transition rate 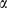 for changes from weak to strong efficacy is set to 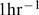, whilst that for changes from strong to weak efficacy, 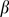, is set to 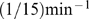. The values of these parameters are chosen (a) to fit the observation that 80% of synapses occupy the weak basal state when the population is in equilibrium [22]; (b) for the model to reproduce data on e-LTP/D decay to good agreement (via decay from the e-LTP/D state followed by equilibration between the two basal states). These rates are comparable with AMPA receptor recycling times [34].

The other strong synaptic states are the e- and 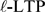 states. They have the same efficacy as the strong basal state, but are considered potentiated states due to their increased resistance to depression. Choosing all potentiated states to have the same weight is motivated by the data which shows that in experiments all LTP forms exhibit very similar amounts of weight change. This is actually surprising given the wide variety of mechanisms that underlie the different forms of LTP. Transitions into the potentiated states only occur during intervals following certain stimulation protocols, which we discuss below. Once a synapse enters the e-LTP state it will decay back into the strong basal state, with transition rate 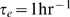, unless it has the opportunity to move into the 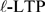 state. The motivation for this decay rate comes from experimental results on e-LTP decay. Furthermore, it is assumed there is a tag present in the e-LTP state since data suggest synapses in an e-LTP state convert to an 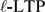 state whenever PRPs become available for capture [12],[13]. Although we do not model the biochemistry explicitly, we suggest that when a synapse is in the e-LTP state, the CaMKII in the synapse is in a phosphorilated state [15].

When a synapse enters the 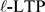 state, it becomes very stable, as the only transition is very slow decay to the strong basal state, with a rate of 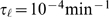. Synapses in the 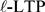 state are assumed to have captured PRPs, such as 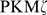 [6],[16]. Although there is some evidence that decay from the 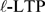 state is an active process rather than passive decay [8],[35], detailed knowledge of this is still lacking, so we did not attempt to include this. The given decay rate is not intended to be precise, but is intentionally of a smaller order of magnitude than the other time-constants of the model. Finally, the model is symmetric in potentiation and depression, and so the LTD states are analogous to the LTP states.

### Transition Rates

The model has ten transitions in total, however setting some rates identical leaves a total of seven transition rate parameters, Figure 1. We have so far mentioned 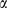 and 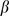 which are responsible for fluctuations between the basal states, as well as 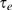 and 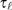 which are the decay rates for e-LTP/D and 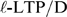 respectively. In addition, there are three further parameters, 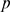, 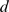 and 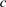, for transitions into e-LTP/D and 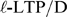 states. These are only switched on following a plasticity-inducing protocol. Note that of these seven parameters, only 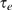 and 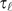 are constant; 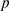, 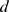, 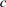, 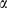 and 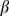 change transiently after stimulation.

### Transition Rates Associated with Early LTP

In this section and the next we discuss the effects of LTP-inducing protocols on the transition rates; the effect on synaptic weight dynamics is discussed in later sections. We model induction in a direct way, focusing on the effects of specific plasticity-inducing stimuli rather than introducing additional stimulus parameters (such as strength, frequency or duration). Specifically, we consider 1) for e-LTP, a single one second burst of HFS (weak HFS); 2) for 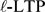, three repeated bursts of HFS, separated by 10 minute time-intervals (strong HFS). The time courses for the transition rates have been chosen so that the model matches the electrophysiological data that the model aims to reproduce.

After any burst of HFS is applied, the following two changes occur. Firstly, the rate 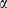 from the weak to strong basal state increases to some very large value for a short period, before returning to its original value. Mathematically, we use 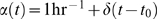 for a stimulus at time 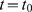. This, in effect, moves all synapses occupying the weak basal state into the strong basal state. This rapid switching is motivated by the above-mentioned observations at the single synapse level [22], and is assumed to come about from AMPA receptor insertion.

**Figure 2:**
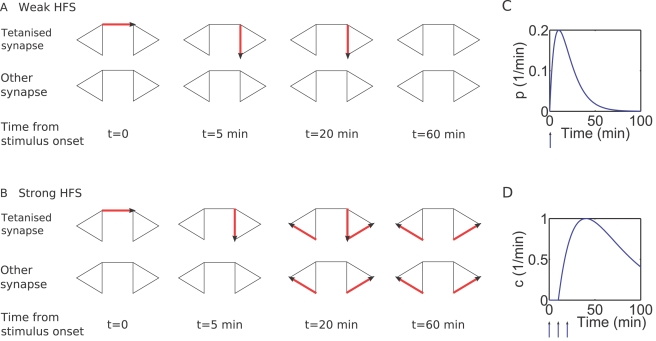
The effects of tetanisation on the state transitions. (A,B) Each diagram represents the state diagram for the model, and the super-imposed arrows indicate the transition rates that are significant in the given scenario, at the given time. To indicate how STC is incorporated into the model, we show a tetanised synapse and an unstimulated synapse. (A) Weak HFS only affects the synapse to which it is applied, and transition into the e-LTP state occurs over a period of a few minutes. (B) Strong HFS initially has the same effect as a weak tetanus. However, protein synthesis and diffusion are triggered by this stimulus: at 20 minutes after stimulus onset, both synapses are affected by rapid transition rates from their e-LTP to 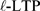 states, and also from their e-LTD to 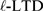 states. Thus if weak HFS or LFS were given to the unstimulated synapse in this scenario, then the STC process would occur. (In the latter case one has “cross-capture”.) (C) The time course of the transition rate 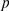 from the strong basal state to the e-LTP state following weak HFS at time 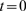. (D) The time course of the transition rate 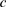 from the e-LTP state to the 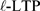 state following strong HFS starting at time 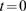.

Secondly, transitions from the strong basal state into the e-LTP state are transiently turned on. Following a stimulus at time 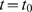, the rate 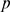 of these transitions is given by an alpha-function 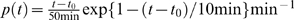. Thus the rate 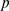 takes a few minutes to grow to a significant level, peaks at a value of 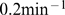, ten minutes after stimulation, and then decays back toward zero, Figure 2. Alpha-functions arise naturally in chemical reaction dynamics. In general, a chain of first-order reactions will lead to a difference of exponentials, while two subsequent reactions with identical rates will yield an alpha-function. Here the alpha-function is assumed to arise from the biochemical induction process in the PSD. The time-course for 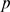 is motivated from evidence that a synaptic tag takes a few minutes to form [36].

Biophysically, the transitions to the e-LTP state might correspond to the phosphorilation of serine-831 of the GluR1 AMPA receptor sub-units during LTP induction [33], which is higher 30 minutes after LTP induction than immediately post-stimulus [2]. Serine-831 phosphorilation is driven by CaMKII phosphorilation which happens on a faster time-scale than that of tag stabilization [36]. A highly simplified model of this cascade would yield an alpha-function. Alternatively, the CaMKII phosphorilation itself might correspond to tag formation and the transition to e-LTP.

### Transition Rates Associated with Late LTP

In addition to evoking the rate changes described above, a synapse subject to strong HFS must incur additional changes resulting from the triggering of protein synthesis and diffusion [4],[5]. This translates in our model to the triggering of the transition rate 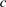 from the e-LTP state into the 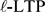 state. As discussed above this might correspond to the capture of 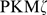. We assume that for 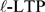 the second burst of HFS crosses the threshold for protein synthesis and the rate 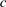 begins to change. Simulations are not sensitive to the precise course 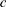 takes, nor is this tightly constrained by experimental data. We assume the plausible form 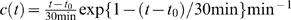 when the second burst of HFS comes at time 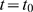. The maximum value of 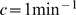 is reached at time 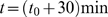. The precise conditions for protein synthesis are not known. The strong HFS protocol described here is not the only protocol that leads to 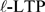; sometimes a strong, single burst of HFS is used [11]. In that instance, we would need to assume that protein synthesis starts sooner. In general, this could be achieved by integrating the stimulation and thresholding it.

The rate 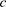 also governs transitions from the e-LTD to the 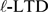 state, which enables the model to describe “cross-capture”, whereby e-LTD of one synapse by weak LFS can be converted to 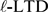 by applying strong HFS to a different synapse [19]. We discuss this further in the section “Modeling synaptic tagging and capture”. Figure 2 summarizes the effects of weak and strong HFS on the transition rates in our model, including their courses.

### Transition Rates Associated with LTD

The effects of LFS are analogous to those of HFS. Both weak and strong LFS affect the rate 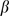 from the strong basal to the weak basal state, and the rate 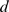 from the weak basal to the e-LTD state in the same way that HFS affects the rates 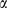 and 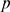, respectively. The only difference is that 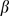 is held very high (at 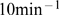) for the extended period of four minutes, to reflect the longer duration of an LFS protocol. The rate 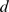 follows the time-course 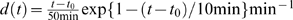, with 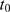 being the time at stimulus onset. This transition could correspond to the de-phosphorilation of serine S-845 [33].

As mentioned above, the rate from the e-LTD state to the 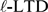 state is given by the same parameter 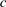 as the rate from the e-LTP state to the 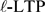 state. Strong LFS triggers this parameter in the same way as strong HFS, i.e., 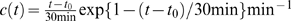 following strong LFS starting at time 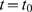. (This can be taken to start at stimulus onset since the strong LFS we consider consists of triple pulses separated by just one second intervals. This is in contrast to our strong HFS protocol, for which the bursts are separated by 10 minute time-intervals.)

### Transition Rates during Synaptic Tagging and Capture

In the above discussion, we have focused on stimulation of a single population of synapses. However STC relates to interactions between different populations of synapses. In our model, transitions from the weak basal state to the strong basal state (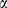), or from the strong basal state to the e-LTP state (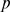) reflect synapse-specific changes; namely changes in the number of AMPA receptors, and configurational changes in the PSD [32],[33]. The transition rates 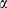 and 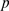 are only modified in stimulated synapses, and hence weak HFS only affects synapses to which it is applied. However, transition from the e-LTP to the 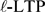 state results in cell-wide changes, i.e., protein synthesis. Thus, after *one* population of synapses has received strong HFS, *many* populations of synapses will see a change in the rate 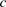 of these transitions. Consistent with experiments, synapses in an unstimulated population have little chance of being in the e-LTP state, and will not be affected by the strong HFS; no tags are present. But if another population of synapses has received weak HFS and move into the tagged (e-LTP) state, then they have a chance to move into the 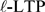 state; proteins are captured by tags. The STC process for LTD is analogous to that for LTP.

Note that there is evidence that the STC interaction has limited range, and can not occur between far away synapses, such as between a basal dendrite synapse and an apical dendrite synapse [15],[37]. In this work we assume that when two different populations within the same neuron are stimulated, they are close enough to interact via STC. However, extension to compartmentalized STC is possible (see Discussion).

As we demonstrate below, the model also accounts for “cross-capture” in a straightforward way by using the same parameter 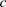 for transitions from the e-LTP state to the 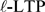 state and from the e-LTD state to the 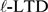 state. Thus, for example, after *one* population of synapses has received strong HFS, synapses from a *second* population that find themselves in the tagged e-LTD state will have a chance to change into the 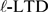 state as a result of LTD tags capturing 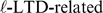 proteins.

### Response of the Model to Plasticity Protocols

#### Modeling physiological LTP and LTD

Next we examine how the model defined above behaves as it is subjected to various plasticity protocols.

**Figure 3:**
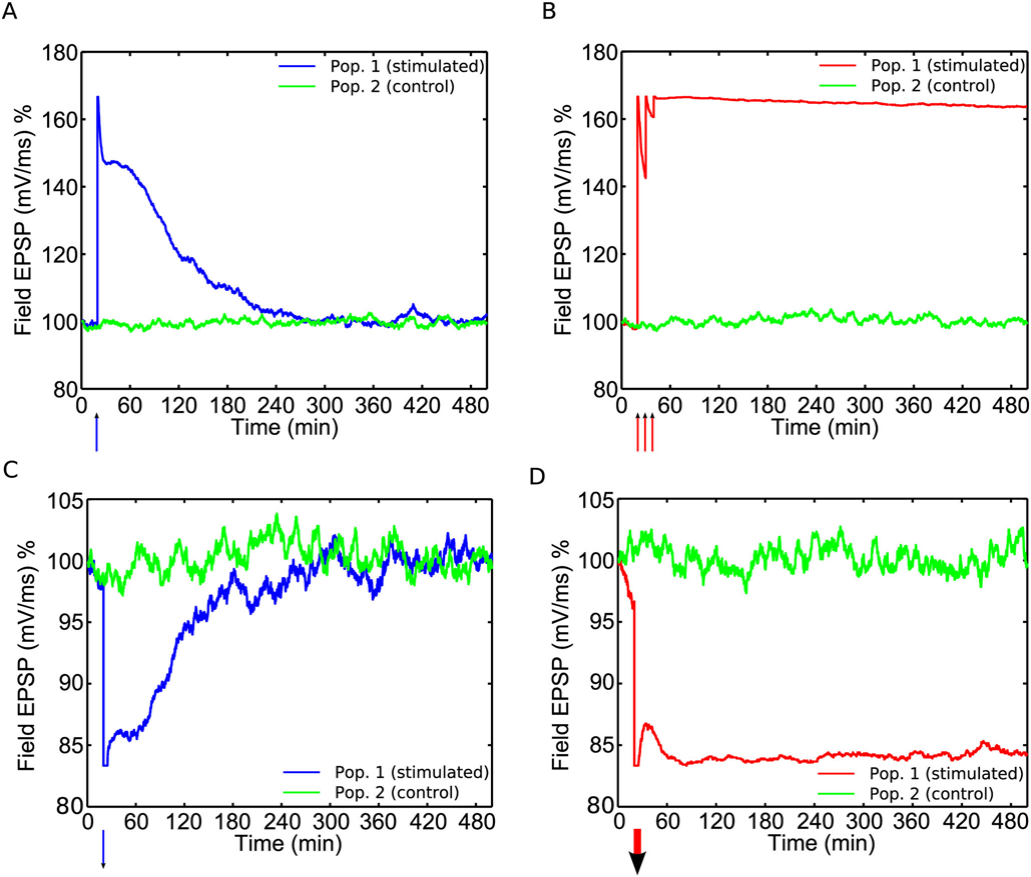
Long-term potentiation and depression in the model. (A) Weak HFS to population 1 at time 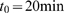 results in e-LTP of that population. The increase in weight to about 150% lasts about 90 minutes. Population 2 is a “control pathway”, that has only test stimulation (to measure its strength) but no tetanic stimulus applied to it. Apart from the fluctuations its weight is stable. (B) Strong HFS to Pop. 1 at 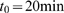 results in 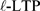 of that population. The control population is not affected. (C) Weak HFS to Pop. 1 at 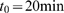 results in e-LTD of that population. (D) Strong LFS to Pop. 1 at 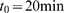 results in 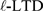.

**Figure 4:**
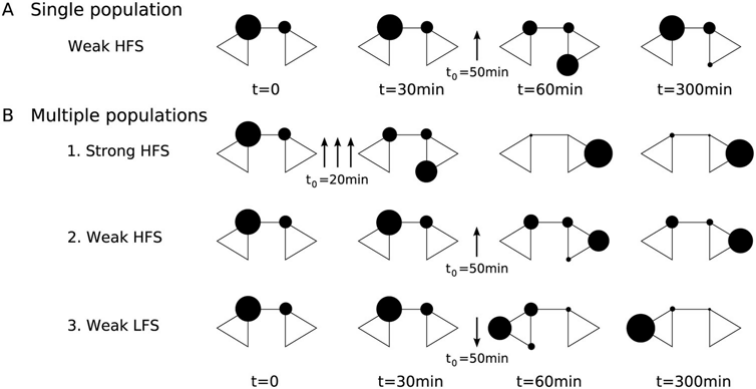
Occupation of states. Each diagram represents the state diagram for the model, and the the area of the circle around each state indicates the proportion of synapses occupying that state. (A) A single population of synapses is given weak HFS at 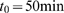. The stimulus results in a transient movement of synapses into the e-LTP state, followed by decay back to the initial state. (B) Multiple populations exhibiting synaptic tagging, capture and cross-capture. One population is given strong HFS at 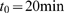. Synapses initially move into the e-LTP state, in which a tag is present, before moving into the 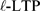 state via the eventual capture of PRPs. A second population is given weak HFS at 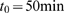. Most of these synapses move swiftly into the 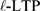 state once the stimulus is given; PRPs are already available as a result of the stimulus to Pop. 1, so capture occurs as soon as tag formation is complete. A third population is given weak LFS at 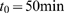. Most of these synapses move swiftly into the 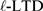 state once the stimulus is given; an LTD tag is set, and this can immediately “cross-capture” 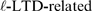 proteins that have been synthesized and diffused as a result of the stimulus to Pop. 1.

As in experiment, weak HFS induces LTP in the target population, and this lasts for around three hours, i.e., the duration of e-LTP, Figure 3A. This comes about from many synapses entering the e-LTP state of the model, where they remain until spontaneous decay to the strong basal state occurs with rate 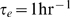. From there, return to equilibrium occurs as synapses fluctuate between the two basal states on the time-scale of 15–30 min, Figure 4A. A control population is plotted alongside the potentiated population, and this is unaffected by the stimulation. Strong HFS leads to long-lasting LTP (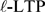), Figure 3B. Synapses move into the 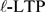 state via the strong basal state and the e-LTP state, and remain stable in this state for a long duration. Figure 3C and 3D show the analogous results for LTD. The lifetime of e-LTD is approximately the same as that of e-LTP. However, the change in fEPSP relative to baseline is smaller for LTD than it is for LTP. This is because in equilibrium 80% of synapses are already in a weak state (weak basal). Thus, on average, only around 20% of synapses can be depressed during LTD, whereas during LTP around 80% of synapses can be potentiated.

#### Synaptic tagging and capture

**Figure 5:**
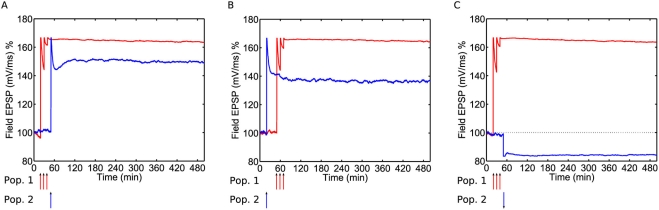
Synaptic tagging and capture in the model. (A) Strong HFS to Pop. 1 at 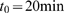 and weak HFS to Pop. 2 at 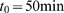 results in 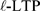 of both populations. (B) Rescue of e-LTP decay: weak HFS to Pop. 2 at 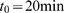 followed by strong HFS to Pop. 1 at 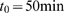. (C) Cross-capture: strong HFS to Pop. 1 at 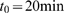 followed by weak LFS to Pop. 2 at 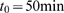.

Figure 5 shows the response of the model to STC protocols. In graph (A) strong HFS to population 1 followed by weak HFS to population 2 leads to 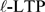 in both populations [12]. The weak HFS to population 2 has caused synapses within this population to move into the e-LTP state. Meanwhile, the strong HFS to population 1 has putatively triggered protein synthesis and diffusion, and this has enabled transitions from the e- to 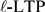 state in both populations of synapses. Thus population 2 synapses migrate further into the 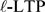 state from the e-LTP state, and we see a prolonged increase in the fEPSP. Weak HFS to population 1 followed by strong HFS to population 2 rescues decay of e-LTP in population 1, Figure 5B, as observed experimentally [13]. Here synapses in population 2 are in the process of decaying from the e-LTP state to the strong basal state, and back into equilibrium, but strong HFS to the other population switches on transitions into the 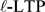 state, and so many synapses are transferred into this state, thus halting decay of the fEPSP. Because of the decay period, the final level of potentiation for population 2 is lower in Figure 5B (weak before strong) than it is in Figure 5A (strong before weak), consistent with data [13].

The model also reproduces “cross-capture”, as observed in [19]. Strong HFS to population 1 followed by weak LFS to population 2 leads to 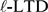 in population 2, Figure 5C. Here the weak LFS to population 2 induces movement into the e-LTD state, but the strong HFS to the other population has enabled synthesis of 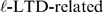 proteins and so further movement into the 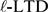 state occurs, resulting in long-lasting depression of the fEPSP. Figure 5 elucidates the STC process further by showing occupancy levels of the states of the model at key times during simulations of (A) weak HFS being given to a single population; (B) multiple populations in which strong HFS is given to one population before two other populations are given weak HFS and weak LFS respectively.

#### De-potentiation

**Figure 6:**
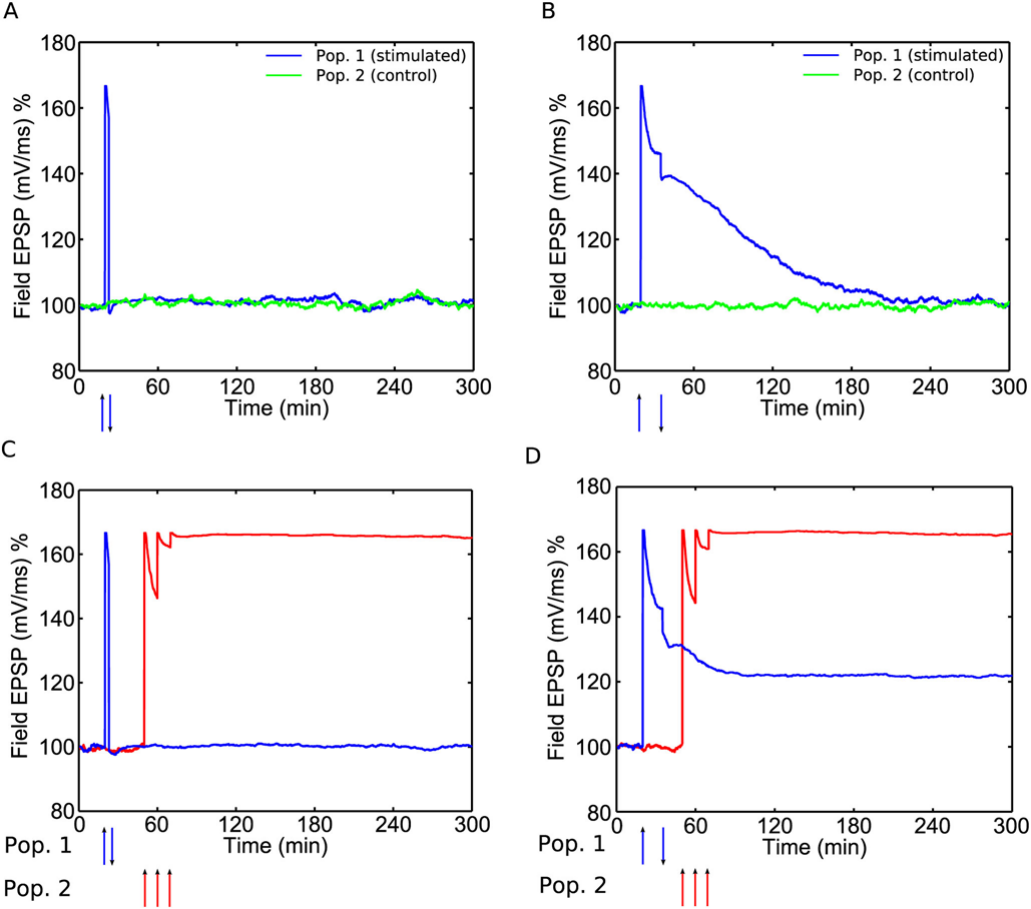
De-potentiation. (A) Weak HFS at 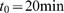 followed by weak LFS at 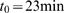 to same population leads to de-potentiation. (B) Weak HFS at 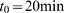 followed by weak LFS at 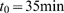. In this case e-LTP is not reversed by the LFS; it has become immune to depotentiation. (C) Pop. 1 is given weak HFS at 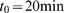 followed by weak LFS at 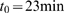, and pop. 2 is given strong HFS at 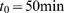. Pop. 1 remains stable at baseline after the stimuli, while pop. 2 exhibits 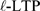. (D) Pop. 1 is given weak HFS at 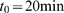 followed by weak de-potentiation LFS at 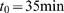, and pop. 2 is given strong HFS at 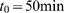. In this instance both populations exhibit 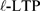.

The model also captures the phenomenon of de-potentiation, in which LTP can be erased by application of an LFS stimulus shortly after the LTP-inducing stimulus. Figure 6A and 6B show that weak HFS followed by weak LFS to the same population leads to no lasting effect if LFS is given three minutes after HFS, but leads to e-LTP if given 15 minutes after HFS, in agreement with data in [36]. This is explained as follows, in terms of state transitions. Immediately after HFS is applied, all synapses occupy the strong basal state, hence the elevated fEPSP. However, movement into the more stable e-LTP state only occurs over a period of a few minutes. Thus, if LFS is given only three minutes after HFS, many synapses are still in the strong basal state, and from there they are moved into the weak basal state by the LFS. This causes the fEPSP to fall back to around 100%. The weakened synapses will mostly move into the e-LTD state, but since some synapses remain in the e-LTP state, the net effect is an un-potentiated fEPSP, which then remains stable as all the synapses fall back into the two basal states. If LFS is administered 15 minutes after HFS, it has little effect since at that stage most synapses occupy the e-LTP state and are immune to de-potentiation.

These results are consistent with data from O'Connor et al. [22] which show de-potentiation of some, but not all, synapses 10 minutes after successful LTP induction. Our model predicts that fewer synapses would de-potentiate, the longer the interval between LTP induction and the LTD protocol. Note that if depotentation is successful, the fEPSP drops back quickly and precisely to the equilibrium baseline value, which would be difficult to explain using continuous instead of binary synapses.

In experiments where immunity to de-potentiation is observed, following the LFS stimulus, the fEPSP drops, but later recovers [36]. This is an effect not seen in our simulations. A possible explanation for this is that the LFS transiently depresses the synapses, masking the decay. Our model does not take pre-synaptic effects into account, which can play a rôle in plasticity on shorter time scales than those of e-LTP and 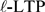 [38].

De-potentiation also interrupts tag formation, thus preventing STC in the de-potentiated population if 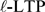 is induced in a second population (see Figure 2 in [36]). Figure 6C shows that our model accounts for this. One population is potentiated and de-potentiated, and then a second population is given strong HFS. Population 2 exhibits 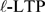 as expected, whilst the fEPSP of population 1 remains stable at around 100%. Due to the de-potentiation, when the strong HFS is administered to population 2 and protein synthesis occurs, very few synapses in population 1 occupy the e-LTP state, and hence there are very few tags present. Thus there can be very little migration into the 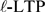 state (STC). Instead the synapses continue to fluctuate between the basal states. Finally, once immunity to de-potentiation occurs, administering LFS does not destroy tags [36]. We reproduce this result in Figure 6D. As in Figure 6B, population 1 is given weak HFS, followed by weak LFS after an interval of 15 minutes. Then population 2 is later given strong HFS and both populations exhibit 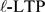. In this instance a good number of population 1 synapses are in the tagged e-LTP state when protein synthesis follows the strong HFS to population 2, and so STC can occur.

### Theoretical Mean and Fluctuation Size

**Figure 7:**
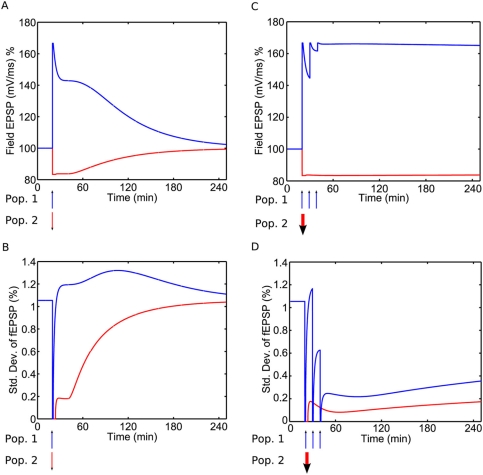
Theoretical mean and fluctuations in the synaptic strength. (A) The time course of the expected value of the fEPSP for a population of 1000 synapses, given either weak HFS (blue, upper curve) or weak LFS (red, lower curve), administered at time 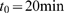. (B) The corresponding standard deviation of the fEPSP as a function of time. The fluctuation increases for e-LTP and decreases for e-LTD. (C,D) Analogous plots for a population given strong HFS or strong LFS; in this case the fluctuations decrease for both protocols.

In addition to reproducing single-trial experiments, the model makes novel predictions about the theoretical mean and inter-trial standard deviation of the fEPSP. Figure 7A and 7B illustrate this for populations of 1000 synapses given weak HFS and weak LFS, whilst graphs C+D illustrate this for strong HFS and strong LFS. We see that when e-LTP is established the standard deviation is greater than at baseline, whilst when e-LTD is established the standard deviation is less, Figure 7B. In the former case, the increase is a result of variability in the number of synapses that make it into the e-LTP state. Although all synapses are initially moved into the strong basal state by the HFS, (resulting briefly in zero fEPSP variability), while the tag-forming reaction in the PSD is still incomplete, a variable number of synapses drop into the weak basal state from where they can no longer access the e-LTP state, Figure 1. Although an analogous process occurs during the onset of e-LTD, the standard deviation remains less in this case since the transition rate from the weak to strong basal state (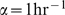) is much less than that from the strong to weak basal state (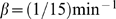). The standard deviation is also less when 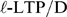 is established, Figure 7D. This is because strong HFS/LFS enables almost all the synapses to enter, first the e-LTP/D state, and then the 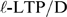 state, in which the weight becomes stable.

The theoretical predictions above can be used in a similar way to the noise analysis technique used to extract properties of voltage- and ligand-gated channels from measurements of their mean current and current fluctuations [39]. In all cases the transition matrix determines not only the evolution of the mean but also the fluctuations around the mean. In principle this means that a more accurate estimate of the transition matrix can be obtained by fitting both the mean and the fluctuations. In analogy with standard noise analysis, here the fluctuations in the basal state are inversely proportional to the number of synapses, the spectrum of the fluctuations can be used to determine the rate constants, and changes to the fluctuations as compared to baseline can be used to calculate how many synapses have made a transition. Although we have attempted to perform this type of analysis on data recorded by Roger Redondo, we found that too many additional noise sources, as well as non-stationarity, makes this analysis currently unsuitable.

## Discussion

We have presented a model of synaptic plasticity at hippocampal synapses which reproduces several slice experiments. It contains just six distinct states, yet gives rise to a rich set of electrophysiological properties. The model incorporates the two observed flavors of LTP and LTD, namely the early and late phases, and de-potentiation, as well as the interaction between these two phases, known commonly as synaptic tagging and capture. The model has a number of key features:

Because all three LTP and all three LTD states have the same weight associated to them (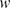 and 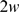, respectively), a given synapse has a binary weight. This is reminiscent of a number of models that have proposed bistable synapses to stabilize memories, often using CaMKII as a switch [25]–[31]. In the current model, synapses have three levels of stability (basal, early-phase and late-phase), with the early- and late-phase being stable up to hours. It is likely that on a biochemical level, bistable switches underlie these more stable states and slow down the transition rates, consistent with those earlier models.

Another key postulate of the model is the existence of a single state that corresponds both to the synapse exhibiting e-LTP and the presence of an 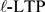 tag, (and similarly for LTD). They go hand in hand; under natural conditions there is no mechanism by which an 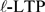 tag can be removed, whilst still retaining e-LTP, or indeed vice-versa, Figure 6. If tag formation is incomplete, de-potentiation (from LFS) can occur and tag formation halted, but if tag formation is complete, de-potentiation can not occur and the tag can not be destroyed, consistent with data in [36]. Pharmacological [15] and genetic manipulations (reviewed in [40]) can interfere with tag setting and capture. The reverse, tag setting without e-LTP, has not (yet) been observed.

Finally, the model makes predictions about the noise level in the fEPSP during a period of potentiation (or depression) followed by a return to baseline value. In particular, it predicts that the noise level increases during a period of e-LTP, but decreases during a period of e-LTD, 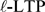 or 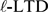, Figure 7. The source of this noise is purely the random nature of the transitions between states. As experimental noise is not taken into account by the model, a test of these predictions would require systematic removal of experimental noise from a data set. The reason for the decreased variability during 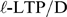 is that many synapses occupy a state that is immune to weight change. An alternative, more complicated model would allow for the possibility of a synapse in a “strong” state to become even stronger, say by insertion of even more AMPA receptors. If this were the case, then a greater level of noise could occur during 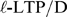 as a result of synapses fluctuating between the 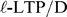 state of our current model and an extra “even stronger” state. Note however that this would be inconsistent with experimental evidence that synapses have only two stable levels of efficacy, e.g. [2],[22].

Next, we discuss shortcomings and potential extensions of the model. In general, it is likely that adding extra states and more complex dynamics would refine the agreement with experimental data. However, doing this incurs the cost of making the model more cumbersome to fit and computationally more expensive. Extra states could, for example, enable us to incorporate the biochemistry of the PSD, leading to a more realistic description of the flow from the basal states into the LTP and LTD states [41]. A recent model of LTP by Smolen [42] indeed incorporates continuous variables for the state of the tag and for protein expression, together with modeling of calcium dynamics.

Protein synthesis probably plays a more subtle rôle in LTP than our model incorporates. For example, immunity to de-potentiation does not require protein synthesis in our model, even though some data suggest it does [43]. Other data suggest that, at high levels of synaptic activation, protein synthesis can be involved in e-LTP as well as in 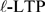 [10]. We have not considered such regimes of reduced protein synthesis in which there could be competition for the capture of proteins available [44]. To reduce the level of protein synthesis, one could simply decrease the post-strong stimulus growth and peak of the transition rate 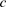, (the rate corresponding to the availability of PRPs). Competition could then be incorporated by reducing the value of 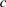 further every time a synapse makes the transition into a 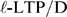 state. Both these effects would reduce the number of synapses that enter the 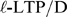 states and the long term change in the fEPSP would be reduced.

Another extension would involve specifying the distances of the site of protein synthesis from the two stimulated populations. Our results are not sensitive to the precise time-course of the transition rate 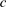, and so our model does not make predictions about this. The time-course for 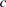 could however be made to reflect the distance of the site of protein synthesis from the stimulated synapses. For very local protein synthesis, 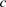 would grow faster and larger than for more distant protein synthesis. In particular, if different populations were at different distances from the site of protein synthesis, then the rate 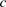 would differ between the two populations. For example, suppose protein synthesis took place near a population of synapses given strong HFS. Then a second population far from this site may only experience STC weakly upon receipt of weak HFS. Few PRPs would be available, so 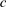 would only grow a little, and only a few synapses would move into the 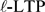 state, causing the stable level of the 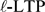 fEPSP to be lower than usual. Such an extension could perhaps account for recent data that suggest that STC interactions do not occur between basal and apical dendrites [15].

Finally, the model does not take into account pre-synaptic effects, which might play a rôle in plasticity on time scales shorter than those of e-LTP and 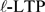 [38]. Extending the model to take account of these could also enhance agreement with data. For instance, in experiments on immunity to de-potentiation one sees a large drop, followed by recovery, in the fEPSP following the application of LFS to an (e-LTP) potentiated population of synapses [36]. In simulations from the model, LTP is also immune, but without this large drop and subsequent recovery, Figure 6B.

Nevertheless, we believe that the model will be useful for continuing theoretical work on the functional consequences of STC, as it captures most known phenomena and is efficient to simulate. In particular it provides a good starting point for neural network modeling. For example, information storage capacity, and the balance between learning and forgetting can be examined for a network of neurons obeying the biophysics of the model. In future theoretical work, this model could be incorporated into a higher-level model that incorporates reinforcement learning and dopamine neurons. It is known that dopamine must be present for 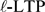 to be established [19],[45]. Moreover, Izhikevich [46] has hypothesized that e-LTP plus tag formation could have the function of maintaining a memory trace of some behavior until a reward signal arrives; upon reward 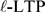 is induced, whilst if there is no reward then the memory trace is lost. Work in these directions is underway.

## Supporting Information

Text S1 List of acronyms
(0.01 MB PDF)
