# Supplementary material for: State Based Model of Long-Term Potentiation and Synaptic Tagging and Capture
Source: PLoS Comput Biol. 2009 Jan 16;5(1):e1000259. doi: 10.1371/journal.pcbi.1000259 (PMC2603667; doi:10.1371/journal.pcbi.1000259)
Supplement: Text S1 — List of acronyms (0.01 MB PDF) [file pcbi.1000259.s001.pdf]

List of acronyms

STC: synaptic tagging and capture; LTP: long-term potentiation; e-LTP: early-phase LTP; ℓ-LTP: late-phase LTP; LTD: long-term depression; e-LTD: early-phase LTD; ℓ-LTD: late-phase LTD; HFS: high-frequency stimulation; LFS: low-frequency stimulation; PRP: plasticity-related protein; PKMζ: protein kinase Mζ; CaMKII: calcium/calmodulin-dependent kinase II; AMPA: α-amino-3-hydroxy-5-methyl-4-isoxazolepropionic acid; PSD: post-synaptic density; fEPSP: field excitatory post-synaptic potential.
